# Update on feline calicivirus: viral evolution, pathogenesis, epidemiology, prevention and control

**DOI:** 10.3389/fmicb.2024.1388420

**Published:** 2024-05-02

**Authors:** Yanquan Wei, Qiaoying Zeng, Huitian Gou, Shijun Bao

**Affiliations:** Department of Preventive Veterinary Medicine, College of Veterinary Medicine, Gansu Agricultural University, Lanzhou, China

**Keywords:** feline calicivirus, evolution, innate immunity, pathogenesis, epidemiology, disease management

## Abstract

Feline calicivirus (FCV) is a prevalent and impactful viral pathogen affecting domestic cats. As an RNA virus, FCV exhibits high mutability and genetic plasticity, enabling its persistence within cat populations. Viral genetic diversity is associated with a broad spectrum of clinical manifestations, ranging from asymptomatic infections and mild oral and upper respiratory tract diseases to the potential development of virulent systemic, and even fatal conditions. This diversity poses distinctive challenges in diagnosis, treatment, and prevention of diseases caused by FCV. Over the past four decades, research has significantly deepened understanding of this pathogen, with an emphasis on molecular biology, evolutionary dynamics, vaccine development, and disease management strategies. This review discusses various facets of FCV, including its genomic structure, evolution, innate immunity, pathogenesis, epidemiology, and approaches to disease management. FCV remains a complex and evolving concern in feline health, requiring continuous research to enhance understanding of its genetic diversity, to improve vaccine efficacy, and to explore novel treatment options.

## 1 Introduction

Feline calicivirus (FCV) is a highly mutagenic RNA virus that is widespread among domestic cats (Zheng et al., 2021). It is host-specific to the *Felidae* family and does not have zoonotic potential. FCV exhibits high genetic and antigenic variability in feline populations (Huang et al., 2010). Clinical manifestations of FCV infection can vary, including upper respiratory tract disease (URTD), lingual ulcerations, gingivostomatitis, limping syndrome, and in severe cases, virulent systemic FCV (*VS*-FCV) infection can cause alopecia, ulceration of the cutaneous, oral cavity, pinnae, nares and necrotizing pododermatitis with serocellular crusts. Other symptoms include subcutaneous edema, bronchointerstitial pneumonia, and pancreatic, hepatic, and splenic necrosis, leading to high mortality in infected cats (Spiri, 2022). Diagnostic methods for FCV include reverse transcription polymerase chain reaction (RT-PCR), virus isolation in cell culture, electron microscopy, immunohistochemistry, and antibody measurements. FCV antibodies can be detected through virus neutralization assays or enzyme-linked immunosorbent assay (ELISA). In cat populations, antibody prevalence is usually high due to natural infections and vaccination. As a result, the presence of specific antibodies is not indicative of an active infection (Bergmann et al., 2019). Treatment primarily involves supportive care, with no licensed antiviral drugs specifically for FCV, however, compounds like nitazoxanide and mizoribine have shown antiviral activity *in vitro* (Cui et al., 2020). Vaccination is crucial in managing FCV, various vaccine types are available, including modified-live and inactivated vaccines (Bordicchia et al., 2021). Hygienic measures and effective disinfection are important in controlling FCV spread, especially in multicat environments (Radford et al., 2009).

## 2 FCV genomic structure

FCV is a highly contagious pathogen of the *Caliciviridae* family, *Vesivirus* genus. This family includes notable human pathogens such as noroviruses, as well as animal-specific viruses like the rabbit hemorrhagic disease virus and the European brown hare syndrome virus. The characteristic cup-shaped depressions on particles are the source of the calicivirus family’s name (Hofmann-Lehmann et al., 2022). FCV carries a single-stranded RNA genome of approximately 7.5 kilobases with a positive sense. The absence of a proofreading mechanism in viral replication contributes to its high mutation rate and thus its rapid evolutionary potential (Vinje et al., 2019). Viral genome is non-segmented and contains three functional open reading frames (ORFs). ORF1 is responsible for the production of a large polyprotein that is subsequently cleaved to generate six non-structural proteins, including an RNA-dependent RNA polymerase (RdRp), which is essential for viral replication (Sosnovtsev et al., 2002). The structural proteins, although encoded in the genomic RNA, are produced from a subgenomic RNA late in the infection. ORF2 encodes a polyprotein that is processed to release a small protein known as the leader of the capsid (LC) and the major capsid protein (VP1). The LC protein, which is unique within the Vesivirus genus, has been identified as essential for the production of viruses capable of inducing a cytopathic effect in feline kidney cell cultures (Abente et al., 2013). VP1 protein forms the majority of virion capsid, ORF3 encodes minor capsid protein VP2 (Cubillos-Zapata et al., 2020).

**Figure 1:**
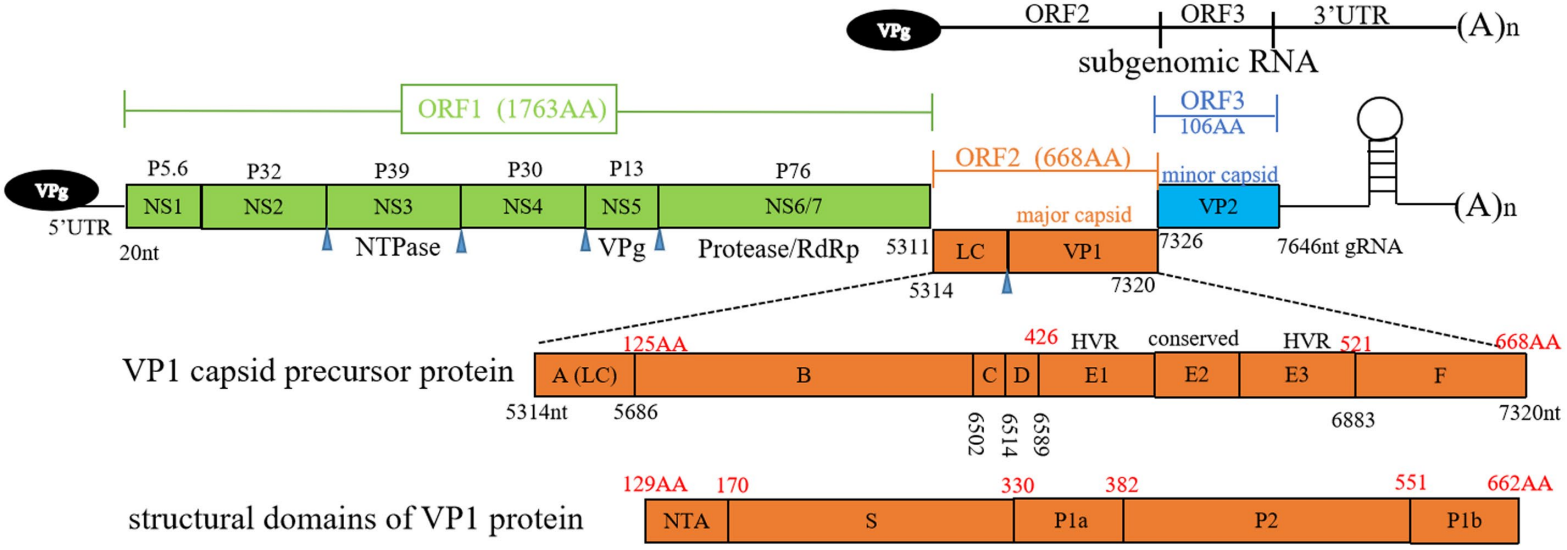
Overview of feline calicivirus (FCV) genomic structure. Open reading frames (ORFs) 1 to 3, subgenomic RNA, antigenic regions (A to F) of VP1 capsid precursor protein, and structural domains of VP1 protein are depicted. Black numbers represent nucleotide positions in viral genome, red numbers represent amino acid positions in ORF2. Blue triangle denotes the cleavage sites of NS6/7. LC, leader of capsid protein; HVR, hypervariable region; RdRp, RNA-dependent RNA polymerase; gRNA, genomic RNA; NTA, N-terminal arm; S domain, shell domain.

The capsid protein VP1 plays a crucial role in facilitating viral entry into host cells, featuring distinct structural domains that include N-terminal arm (NTA), shell (S), and protruding domain (P), which is further divided into subdomains P1 and P2, as depicted in Figure 1. Within VP1 protein, antigenic regions A to F, and especially region E, are known to engage with viral receptor, feline junctional adhesion molecule A (fJAM-A) (Figure 1). fJAM-A is located at cellular tight junctions, and its disruption is associated with formation of oral and cutaneous ulcers. Recent investigation has revealed that FCV employs clathrin-mediated endocytosis as a pathway for cellular entry, additionally, acidification of endosomes is necessary for the uncoating of viral genome (Stuart and Brown, 2006).

The capsid protein VP2 is essential for the uncoating of FCV, facilitating release of the viral genome within host cells (Conley et al., 2019). Interaction between VP1 and VP2 is critical for the structural integrity and function of viral particle, some linear epitopes recognized by neutralizing and non-neutralizing antibodies are identified in different regions of VP1 (Cubillos-Zapata et al., 2020). Antigenic variations among FCV isolates present challenges for achieving vaccine cross-protection. However, the degree of identity among strains permits a certain level of cross-protection (Yang et al., 2023).

## 3 Viral evolution

FCV is known for its high mutation rate, displaying significant evolutionary dynamics both within individual hosts and across populations. The annual rate of nucleotide substitutions for FCV ranges from 1.32 × 10^−2^ to 2.64 × 10^−2^ within individuals and from 3.84 × 10^−2^ to 4.56 × 10^−2^ among populations (Coyne et al., 2007). This places FCV among the RNA viruses with the one of highest identified evolution rates. Sequence analyses reveal significant genetic heterogeneity among closely related isolates, indicating the existence of FCV as a quasispecies within the host. Through analysis of nucleotide and amino acid sequences within HVRs, FCV has been classified into two genogroups, with genogroup II isolates exclusively originating from Japan (Sato et al., 2002).

Studies on FCV genetic diversity and viral evolution propose a 20% genetic distance as the threshold for strain differentiation. Isolates that are epidemiologically related, particularly those associated with outbreaks of virulent systemic disease, typically demonstrate approximately 99% identity, this high level of identity indicates variants of the same strain. Within FCV-endemic cat colonies, there can be up to 18% viral variation within a single strain. FCV strains exhibit notable genetic and antigenic complexity at both spatial and temporal levels, and no single field strain tends to dominate in these populations (Coyne et al., 2012). Viral evolution in FCV is not solely driven by competition among different isolates; long-term survival within a susceptible population involves progressive accumulation of random mutations, sequential reinfection, and recombination. These strategies contribute to increased genetic variability, potentially leading to the emergence of new strains and enable endemic infections (Coyne et al., 2007).

## 4 Innate immunity during FCV infection

Interferon Regulatory Factor 1 (IRF-1) was found to significantly inhibit FCV replication. IRF1, localized in the nucleus, efficiently activates interferon (IFN) beta and the interferon-stimulated response element (ISRE) promoter. It can also trigger the expression of interferon-stimulated genes (ISGs). The mRNA and protein levels of IRF1 were reduced following FCV infection, which may be a new strategy for FCV to evade the innate immune system (Liu et al., 2018).

The FCV strain 2,280 exhibits resistance to IFN-β. The molecular mechanism involves the viral ability to block the JAK–STAT pathway by facilitating the mRNA degradation of IFN alpha and beta receptor subunit 1 (IFNAR1), which is mediated by the FCV P30 protein. An *in vitro* degradation assay revealed that the P30 protein from strain 2,280, but not from the vaccine strain F9, has the capacity to directly and selectively degrade IFNAR1 mRNA. Furthermore, a reverse genetic system used to exchange the P30 proteins between the 2,280 and F9 strains has revealed that the P30 protein from strain 2,280 plays a pivotal role in conferring resistance to IFN and enhancing the viral pathogenic potential (Tian et al., 2020). Another FCV protein, P39, was found to suppress the production of IFN-β and ISG15 mRNA. P39 expression also inhibited the phosphorylation and dimerization of IRF-3, furthermore, P39 inhibits the production of type I IFN by preventing IRF-3 activation (Yumiketa et al., 2016). FCV infection initiates autophagy, and the non-structural proteins P30, P32, and P39 are responsible for this process. Increased autophagy further suppresses FCV-induced Retinoic Acid Inducible Gene I signal transduction, indicating that autophagy can modify the innate immune response to FCV infection (Mao et al., 2023).

## 5 Epidemiology

FCV primarily affects cats, with no known reservoirs or alternative hosts except for wild felids. Humans are not susceptible to infection. FCV-like viruses have sporadically been isolated from dogs, and their role in the epidemiology of FCV in both dogs and cats is uncertain. The FCV strain TIG-1 was isolated from the feces of a Siberian tiger in 2014. The growth kinetics of strain TIG-1 demonstrated a more rapid increase in viral load compared to F9 strain between 12 and 36 h post-infection. TIG-1 is identified as a highly virulent FCV strain, causing 100% morbidity and lethality in infected cats (Tian et al., 2016). Shedding of FCV is primarily through oral and nasal secretions in cats with acute disease, but it is also present in their blood, urine, and feces. Cats may continue shedding for at least 30 days post-infection, with a few shedding for several years or even life-long. A subset of cats seems resistant to infection despite continuous exposure, likely due to immune-mediated mechanisms or genetic factors. FCV is detected in both cats with acute infections and in those that have seemingly recovered yet remain carriers. The viral ability to persist in the environment is significant, as it can survive on dry surfaces at room temperature for days to weeks, and even longer in colder, more humid conditions (Nims and Plavsic, 2013).

Immune condition hinders FCV infection to a certain degree. While pre-existing immunity, either from maternally derived antibodies (MDA) or vaccination, can lessen or eliminate clinical symptoms, it does not prevent infection, and vaccinated or naturally immunized cats can still become carriers after sub-clinical infection, this highlights the role of silent carriers in epidemiology of the disease. Shelters with high turnover demonstrate higher genetic variability of FCV compared to more stable multicat households (Pereira et al., 2018). Indirect transmission is a concern in catteries, shelters, and veterinary clinics, with potential spread via fomites, people, aerosols, or even flea feces (Spiri et al., 2019).

## 6 Pathogenesis of FCV

FCV has been a valuable model for studying molecular pathogenesis, benefiting from its cultivability in cell culture, unlike other members of the *Caliciviridae* family (Spiri, 2022). It has served as an essential organism for exploring viral pathogenesis and disinfection methods. FCV infection in cats primarily occurs in the oropharynx, causing transient viremia, which spreads FCV to various tissues. Immunohistochemistry and electron microscopy have confirmed that *VS*-FCV infects feline epithelial and endothelial cells, leading to cell death, vascular damage, and high mortality (Pesavento et al., 2004). *VS*-FCV strains target fJAM-A, a receptor found at epithelial and endothelial cell junctions, causing junctional disruption and leakage (Pesavento et al., 2011). Additionally, the presence of the fJAM-A receptor on feline platelets and blood leukocytes suggests a hematogenous distribution of FCV, as indicated by the detection of FCV RNA in the blood (Pesavento et al., 2011). Introduction of a fJAM-A expression system into non-permissive cells has been found to convert them into permissive cells for FCV, highlighting the essential receptor role of the fJAM-A proteins (Makino et al., 2006).

In addition to the typical oral symptoms, FCV can also cause lameness, a condition known as limping syndrome. The development of the limping syndrome is associated with immune complexes, and FCV can be found in the affected joints. Polyarthritis, characterized by lameness and fever, can occur in cats following FCV infection or vaccination, and has been hypothesized to be a type III hypersensitivity reaction (Hartmann et al., 2023). In this reaction, antigen–antibody complexes form and accumulate in the joints, triggering an acute inflammatory response. While the syndrome has been linked to FCV, co-infections with FCV field strain or, exceptionally, vaccine strain reactivation, are considered the primary triggers (Dawson et al., 1994). Infected cats exhibit elevated levels of cytokines, especially IL-10, TNF-alpha, and MIP-1alpha, indicating a systemic inflammatory response (Foley et al., 2006).

In cell culture, FCV infection elicits a distinctive cytopathic effect characterized by cell rounding and membrane blebbing (Fumian et al., 2018). Molecular research has shown that FCV infection in cell culture activates the mitochondrial pathway, which, in turn, triggers the activation of caspases and apoptosis (Natoni et al., 2006). To evade host’s defenses, FCV employs various strategies, including antigenic drift and shift. FCV’s error-prone RNA polymerase results in the continuous accumulation of mutations (antigenic drift), and recombination events between distinct FCV strains (antigenic shift) have also been documented. These changes can lead to significant modifications in viral epitopes, which may reduce recognition by neutralizing antibodies or enhance FCV’s ability to bind to cell receptors, thereby increasing its infectivity. Another of the immune evasion strategies used by FCV involves inhibiting host protein synthesis, a process known as host “shut-off” (Wu et al., 2016). This mechanism restricts the host’s protein synthesis to only the essential proteins required for viral replication, thereby compromising the host’s antiviral capability (Wu et al., 2021).

## 7 Detection and diagnosis of FCV infection

Detection and diagnosis of FCV infection involve a range of methods and viral genome can be detected through a variety of PCR-based assays, such as conventional, nested, and real-time RT-PCR. Despite assay optimization, some established RT-PCR systems may fail to amplify all FCV isolates due to the viral high genetic variability (Meli et al., 2018). Multiplex RT-PCR assays, which detect FCV alongside other pathogens, may be less sensitive. Real-time RT-PCR is generally preferred for its higher sensitivity, and quantitative assays can provide information about the viral load. Swabs from the oropharynx and tongue are more likely to yield positive results compared to conjunctival swabs (Schulz et al., 2015). A rapid and robust detection method, enzymatic recombinase amplification coupled with a lateral flow dipstick, shows potential but requires further validation (Liu et al., 2022). While a colloidal gold immunochromatographic assay has been successfully developed for detection of human noroviruses, using shell domain of VP1 protein (Xu et al., 2021), the application of this assay for FCV detection has not yet been reported.

Virus isolation is a valuable method for detecting FCV infection; however, it may not always be successful due to several factors, including the need for sufficient sample quantity, the potential for virus inactivation during transport, and interference from antibodies present in the sample. FCV can be isolated from swabs taken from the oropharynx, nose, or conjunctiva. The sensitivity of detection can be enhanced by combining RT-PCR with virus isolation methods. A negative test result does not definitively rule out infection, especially in suspected cases (Meli et al., 2018). Electron microscopy and immunohistochemistry are potent diagnostic tools but are not practical for routine use due to their specialized equipment and technical requirements (Monne Rodriguez et al., 2014). Immunohistochemistry can be used to confirm the presence of FCV particles. In cats naturally infected with *VS*-FCV, viral antigen was detected within the endothelial and epithelial cells of affected tissues through immunohistochemical staining with an FCV-specific monoclonal antibody (Pesavento et al., 2004). Furthermore, in a case of polyarthritis associated with FCV infection, the synovial membranes were also immunohistochemically stained with a mouse monoclonal antibody targeting the FCV capsid protein (Balboni et al., 2022). Antibody detection methods, such as virus neutralization, ELISA, or immunofluorescence, are not ideal for diagnosing acute infection, because high levels of antibodies are prevalent in the population, and these tests lack the specificity needed to distinguish between current and past infections (Bergmann et al., 2019). Diagnosis of *VS*-FCV infections does not rely on specific genetic markers. Instead, it requires a comprehensive assessment of clinical signs, epidemiological context, and molecular diagnostic findings. To date, no single laboratory test can differentiate between classical FCV and *VS*-FCV infections (Hofmann-Lehmann et al., 2022). The current detection methods reported in the literature are summarized in Table 1.

**Table 1:** Detection and diagnostic methods for FCV infection.

Detection target	Assay type	References
Nucleic acids	(Triplex) TaqMan real-time reverse transcription-PCR	Abd-Eldaim et al. (2009), Cao et al. (2021)
	(Multiplex) RT-PCR	Scansen et al. (2005), Kim et al. (2020)
	RT-qPCR (or with fluorescence resonance energy transfer probe)	Helps and Harbour (2003), Wilhelm and Truyen (2006), Meli et al. (2018), Phongroop et al. (2023)
	CRISPR-Cas13a based visual detection	Huang et al. (2022)
	Enzymatic recombinase amplification combined with a lateral flow dipstick	Liu et al. (2022)
	Multiplex RT-PCR/PCR	Sykes et al. (2001)
	Dual/triple Nano-PCR	Ye et al. (2022), Yan et al. (2023)
Virus particles	CrFK cell-based plaque assay	Bidawid et al. (2003)
	Carboxymethyl-cellulose plaque assay	Escobar-Herrera et al. (2007)
Antigens or antibodies	Immunohistochemistry	Pesavento et al. (2004), Balboni et al. (2022), Fontes et al. (2023)
	Enzyme-linked immunosorbent assay (ELISA) for antigens	Tajima et al. (1998)
	Point-of-care ELISA for protective antibodies	Digangi et al. (2011)
	Double-antibody sandwich ELISA for antigens	Yuan et al. (2014)
	Surface plasmon resonance biosensor for antigens	Yakes et al. (2013)

## 8 Management of FCV infection

### 8.1 Treatment of FCV infection

FCV, lacking an envelope of phospholipid bilayers, exhibits high tenacity. RT-qPCR has detected FCV RNA in the environment of infected cats up to 28 days after shedding stopped, though no replication-competent virus was detected at any time (Spiri et al., 2019). In shelters and animal hospitals, the constant intake of new cats with unknown immune, vaccine, and disease backgrounds, combined with FCV’s prolonged environmental stability, poses a substantial risk. Therefore, hygiene and disinfection are critically important, including thorough cleaning of all surfaces, followed by the use of disinfectants known to be effective against FCV, such as sodium hypochlorite (2,700 ppm, 1 min), accelerated hydrogen peroxide (35,000 ppm, 10 min), aldehydes (2%), or potassium peroxymonosulfate (1%, 10 min) (Chiu et al., 2015). Virucidal disinfectants effective against human norovirus are also suitable for FCV, given the similar viral properties of these two pathogens (Malik and Goyal, 2006). Treatment of cats with URTD caused by FCV focuses on supportive care, which includes administration of intravenous fluids, non-steroidal anti-inflammatory drugs, nebulization therapy, and provision of highly palatable food to maintain nutrition (Radford et al., 2009).

Recombinant feline interferon omega (feIFN-ω) has shown efficacy in reducing clinical signs and FCV replication (Matsumoto et al., 2018). Although no direct antiviral drugs against FCV are currently available on the market, research has investigated potential treatment options. The combination of feIFN-ω and mefloquine has shown promise in inhibiting FCV replication. Nitazoxanide and mizoribine have demonstrated antiviral activity both *in vitro* and *in vivo*, suggesting potential therapeutic benefits (Cui et al., 2020). Drug screening has identified handling as an inhibitor of FCV infection through its suppression of HSP70 expression *in vitro* (Yan et al., 2024). Several antiviral agents, including ribavirin, polysodium 4-styrenesulfonate, mefloquine, and natural compounds, have demonstrated inhibitory effects against FCV replication *in vitro*; however, their efficacy *in vivo* is still uncertain (Hofmann-Lehmann et al., 2022). Sera containing antibodies against FCV, feline herpesvirus (FHV), and feline parvovirus (FPV) have shown efficacy in the treatment of acute viral feline URTD (Friedl et al., 2014).

In the treatment of feline chronic gingivostomatitis (FCGS) caused by FCV infection, a variety of approaches have been explored, including dental hygiene, corticosteroid therapy, and mesenchymal stem cell therapy. Broad-spectrum antibiotics are prescribed for FCGS when secondary bacterial infections are present. Prednisolone and felFN-ω are considered for managing FCGS, moreover, reducing stress, enriching the environment, and maintaining good hygiene are essential in preventing FCGS in multi-cat households (Hennet et al., 2011).

### 8.2 Vaccination

Vaccination is a critical component of FCV infection management, providing protection against severe clinical signs, inflammation, and reducing viral shedding. Both modified-live and inactivated vaccines are commercially available (Ford, 2016). Emerging vaccine technologies, such as mRNA vaccines and subunit vaccines, hold promise but are not yet commercially accessible. Vaccination guidelines take into account factors like age, health status, lifestyle, and housing conditions to determine the appropriate vaccination intervals and types (Ford, 2016).

The emergence of FCV shedding and the onset of clinical symptoms were accompanied by a surge in anti-FCV IgM antibodies (Knowles et al., 1991). This initial rise in IgM levels was quickly succeeded by a rapid escalation of IgG antibodies, which coincided with the initiation of the virus-neutralization activity. This evidence suggests that IgG antibodies are the primary contributors to the neutralizing capacity, while IgM antibodies appear to be essential in the early phase of the neutralizing response. However, it’s challenging to distinguish between vaccine-induced and wild-type antibodies. Additionally, studies have investigated the presence of IgA antibodies in both saliva and serum, observing that the peak levels of IgA antibodies were detected earlier in saliva than in serum, indicating a potential role for salivary IgA in the early defense against FCV (Spiri, 2022). Traditional vaccination schedule suggests primary doses at 8–9 and 12 weeks of age, followed by annual boosters. The effectiveness of this schedule is a subject of debate, and evidence suggests that not all kittens develop adequate immunity by 12 weeks due to lingering MDA. The duration of immunity can vary, particularly against heterologous challenges. Antigenic diversity among FCV strains can result in occasional vaccine failures, so the improving trend of vaccines involves enhancing cross-reactivity, reducing challenge virus shedding, and preventing persistent infection. The lack of biomarkers for protection makes challenge experiments essential for assessing vaccine efficacy (Radford et al., 2007).

FCV vaccines are often combined with those for FHV or FPV. Inactivated vaccines containing adjuvants may carry the risk of inducing injection-site sarcomas. However, a non-adjuvanted, inactivated vaccine against FCV, which is formulated using a combination of two killed virus strains, is available in Europe and Japan. The use of modified-live vaccines is also a point of consideration because they may lead to the circulation of FCV strains, potentially contributing to the emergence of immune-evasive variants. The value of antibody testing in predicting protection is limited, as it may not correlate with actual field exposure. Vaccination of recovered healthy cats is recommended. The vaccination strategy differs for kittens and older cats, taking into account MDA and risk factors. The optimal revaccination intervals are debated, with suggestions ranging from triennial boosters in low-risk situations to annual boosters in high-risk environments (Hofmann-Lehmann et al., 2022).

## 9 Conclusions and prospectives

FCV has been a significant pathogen in cats for over four decades, and its persistence in the feline population is associated with ongoing evolutionary processes. With its broad prevalence, FCV serves as a valuable model for studying the effects of virus evolution within its natural host. FCV is not only a major cause of URTD in cats, but it is also increasingly recognized as a source of virulent systemic disease. The emergence of highly virulent strains emphasizes the urgency of improving the cross-protection afforded by vaccines. As FCV continues its evolution, strategies for its control must be consistently updated.

Rapid identification of the disease through recognition of clinical signs and use of appropriate testing is essential. The diagnosis of FCV infection requires a comprehensive approach, taking into account clinical, molecular, and epidemiological factors for accurate interpretation. Managing FCV infection necessitates a multifaceted strategy, involving supportive care, potential antiviral therapies, and preventive measures. Rigorous enforcement of disinfection, isolation, and quarantine measures is crucial to reduce mortality of FCV infection. Although significant progress has been made in understanding treatment options, further research is imperative to develop effective therapies for FCV-related diseases.

## Author contributions

YW: Writing – original draft, Writing – review & editing. QZ: Writing – original draft, Writing – review & editing. HG: Supervision, Writing – original draft, Writing – review & editing. SB: Supervision, Writing – original draft, Writing – review & editing.
